# Clinical outcomes of long follicular-phase protocol in first-cycle patients with diminished ovarian reserve and AMH<1.2 ng/mL: A single-center study

**DOI:** 10.1097/MD.0000000000047609

**Published:** 2026-02-13

**Authors:** Fang Hong, Huaying Yu, Feng Zhou, Xiaomei Tong

**Affiliations:** a Center of Reproductive Medicine, Sir Run Run Shaw Hospital, School of Medicine, Zhejiang University, Hangzhou, China; b Key Laboratory of Reproductive Dysfunction Management of Zhejiang Province, Department of Obstetrics and Gynecology, Hangzhou, Zhejiang, China.

**Keywords:** AMH, diminished ovarian reserve (DOR), GnRH antagonist protocol, long follicular-phase protocol, progestin-primed ovarian stimulation protocol (PPOS)

## Abstract

This study aims to compare the outcomes of assisted reproductive treatment in women with low ovarian reserve (LOR), defined as an anti-Müllerian hormone level of ≤1.2 ng/mL, versus normal ovarian reserve (NOR; 1.2 ng/mL < anti-Müllerian hormone ≤3.0 ng/mL) using the long follicular-phase protocol. It further evaluates the efficacy of the long follicular-phase, antagonist, and progestin-primed ovarian stimulation (PPOS) protocols specifically within the LOR population. A retrospective analysis was conducted on 2309 patients treated between October 2022 and October 2024. Participants included 973 LOR patients and 1336 NOR patients. LOR patients were divided by protocol: Group A (long follicular-phase protocol, n = 95), Group C (antagonist, n = 200), and Group D (PPOS, n = 678). All NOR patients received the long follicular protocol (Group B, n = 1336). Groups were stratified by age (<35 and ≥35 years). Regarding the long follicular protocol: In women <35, Group A had significantly fewer retrieved oocytes and high-quality embryos than Group B (*P* <.05), yet clinical pregnancy and live birth rates (LBRs) were similar (*P* >.05). In women ≥35, Group A had fewer oocytes but a higher MII rate than Group B, with no significant difference in pregnancy or live birth rates. Comparing protocols within the LOR population: Group A exhibited significantly lower baseline hormone levels but higher numbers of punctured follicles, retrieved oocytes, and 2PN fertilized oocytes compared to Groups C and D (*P* <.05). Notably, among patients ≥35, Group A produced significantly more high-quality embryos. Generally, age negatively impacted outcomes across all groups. For individuals with LOR, the long follicular-phase protocol yields clinical pregnancy and live birth rates comparable to those with normal reserve, despite lower oocyte yield. Our research findings, while not conclusive in the LOR population, suggest a possible trend that long follicular-phase protocol may offer certain advantages over antagonist and PPOS protocols in key experimental parameters. Nevertheless, age remains a key factor affecting stimulation response.

## 1. Introduction

“Ovarian reserve” refers to the number of remaining oocytes in the ovary. Whether the early decline of ovarian reserve is only manifested as a decrease in the number of oocytes or accompanied by a decline in quality remains controversial. The number and quality of oocytes belong to different concepts. The latter specifically refers to the intrinsic ability of oocytes in aspects such as fertilization, maintaining embryo development, and producing euploid embryos, and is significantly correlated with female age.^[1–3]^ Among women of similar ages, individual differences in oocyte numbers are evident. Research on the link between ovarian reserve and oocyte quality reveals significant discrepancies. Notably, there is no consensus on how diminished ovarian reserve (DOR) relates to the risk of aneuploid pregnancies.^[4,5]^ Some studies suggest that reduced levels of ovarian reserve markers, such as AMH, correlate with higher rates of embryo aneuploidy and clinical miscarriages,^[6]^ indicating a potential mechanistic link between follicle number and quality. However, in young women with DOR, no association is found between DOR and increased aneuploidy incidence.^[7,8]^ Another study reported that, after adjusting for age, euploidy rates in patients with low and normal ovarian reserves (NORs) are comparable.^[9–12]^ Conversely, some studies indicate that even after age adjustment, women with DOR have lower blastocyst euploidy rates compared to non-DOR women, and this is related to the number of retrieved oocytes.^[13–15]^

In assisted reproductive technology (ART), clinical practice typically employs indicators like follicle-stimulating hormone (FSH), antral follicle count (AFC), AMH and age to assess ovarian reserve. AMH has increasingly supplanted FSH as the preferred marker due to its distinct advantages. The clinical applications of FSH is constrained by significant inter-cycle variability and the necessity for concurrent evaluation with estradiol (E2) levels. In contrast, AMH offers low intra- and inter-cycle variability, providing a more reliable prediction of ovarian response to gonadotropins.^[16,17]^ Additionally, unlike AFC, which is highly dependent on the operator’s expertise and equipment quality, AMH is more advantageous for assessment. Despite technical challenges such as kit standardization and sample storage stability,^[18,19]^ AMH remains the superior choice. Consequently, AMH was selected for ovarian reserve evaluation in this study.

In current clinical practice, biomarkers associated with ovarian reserve (e.g., AMH) are predominantly used to forecast the ovarian response to controlled ovarian stimulation (COS) in ART, but their value in predicting embryological outcome and live birth rate (LBR) is still controversial,^[20]^ especially in the low-reserve population of DOR. As a special population in assisted reproduction, low-reserve population often faces the problem of less oocyte acquisition, and whether the oocyte development potential is damaged and the influence of different ovulation promotion protocols on their pregnancy outcomes need to be further explored after excluding age factors. For normal-reserve patients, the GnRH agonist-based follicular-phase long protocol is considered a standard regimen due to effective LH suppression and stable follicular synchronization.^[21]^ However, its use in DOR is controversial: while traditionally thought to worsen poor response via pituitary over-suppression,^[22]^ recent observations suggest that optimized dosing may still offer acceptable outcomes in selected younger, less severely affected patients.^[23]^ Thus, whether this protocol can achieve satisfactory pregnancy results in DOR remains a key unanswered clinical question.

Based on this, this retrospective study examines women undergoing in vitro fertilization–embryo transfer (ET) (IVF-ET), with emphasis on low ovarian reserve (LOR), to assess the relationship between ovarian reserve – quantified by serum AMH – and oocyte quality indicators. The study has 2 parts. First, it compares clinical outcomes across patients with different AMH levels who were treated with the long follicular-phase protocol. Second, it compares the ovarian stimulation effects of different ovulation induction protocols among patients with DOR. The research aims to determine the independent effect of DOR on oocyte development and to evaluate whether the long follicular-phase protocol improves pregnancy outcomes in this population, thereby informing individualized clinical decision-making.

## 2. Materials and methods

### 2.1. Patient selection

We collected data from patients who underwent in vitro fertilization/intracytoplasmic sperm injection–ET (IVF/ICSI-ET) at the Reproductive Center of Sir Run Run Shaw Hospital, Zhejiang University School of Medicine, between October 2022 and October 2024. Based on AMH levels, the cohort comprised 973 patients with LOR (AMH ≤1.2 ng/mL) and 1336 patients with NOR (1.2 ng/mL < AMH ≤3.0 ng/mL). AMH was measured for all patients within 6 months before initiation of ovulation induction. The study was approved by the hospital Ethics Committee (SRRSH-IRB-2025-Study-No.1212).

### 2.2. The inclusion criteria are as follows:

1. Infertile women aged 20 to 45 years;
2. Regular menstrual cycles (21–35 days) before stimulation;
3. Body mass index (BMI) between 18.0 and 30.0 kg/m^2^

(exclusive of boundary values);

1. No history of IVF or ICSI treatment;
2. No history of chemotherapy or pelvic radiotherapy.
3. No history of any ovulation induction therapy, hormonal medication (e.g., oral contraceptives, glucocorticoids) within 3 months prior to enrollment.

### 2.3. Exclusion criteria:

Included the presence of any of the following conditions:

1. Both spouses suffer from genetic diseases, congenital malformations, recurrent miscarriages, or have a history of cancer.
2. There are irreparable uterine abnormalities or malformations that may interfere with embryo implantation.
3. Both spouses suffer from systemic diseases (e.g., autoimmune diseases, diabetes).
4. Embryos cryopreserved after preimplantation genetic testing for aneuploidy (PGT-A).

### 2.4. Grouping

Patients with LOR were divided into 3 groups according to the ovarian stimulation protocol: Group A (long follicular-phase protocol, 95 cases), Group C (antagonist protocol, 200 cases), and Group D (progestin-primed ovarian stimulation [PPOS] protocol, 678 cases). Patients with NOR all received long follicular-phase protocol (Group B, 1336 cases). Each group was further divided into 2 subgroups based on age: the <35 years subgroup (A1, B1, C1, D1) and the ≥35 years subgroup (A2, B2, C2, D2).

### 2.5. Protocols for ovarian stimulation

#### 2.5.1. Long follicular-phase protocol

On menstrual cycle days 2 to 3, a long-acting gonadotropin-releasing hormone agonist (GnRH agonist; leuprorelin acetate microspheres for injection, 3.75 mg; Livzon Pharmaceutical Group Inc., Zhuhai, China) was administered o achieve pituitary down-regulation. After 30 to 42 days, down-regulation was confirmed by transvaginal ultrasound showing no ovarian cysts >10 mm in diameter and serum hormone levels meeting the following criteria: estradiol (E2) <30 ng/L (183 pmol/L) and luteinizing hormone (LH) <3 IU/L. Ovarian stimulation was then initiated with recombinant FSH (rFSH) or human menopausal gonadotropin at a daily dose of 150 to 300 IU, determined individually based on the patient’s age, BMI, AMH level, basal FSH, and AFC. The gonadotropin dose was subsequently adjusted according to serial monitoring of follicular size, number, and serum E2 levels until the leading follicles reached maturity, meeting the criteria for trigger injection.

#### 2.5.2. GnRH antagonist protocol

Ovarian stimulation was initiated on menstrual cycle day 2 to 3. Based on the patient’s age, BMI, AMH, basal FSH and AFC levels, the individualized starting dose of gonadotropin was determined to be 150 to 300 IU per day. An antagonist was added using a flexible protocol. Subcutaneous ganirelix acetate (GnRH antagonist; Ganirelix Acetate Injection, 0.25 mg/prefilled syringe; Organon, Oss, the Netherlands) was administered once daily when the dominant follicle diameter reached ≥14 mm or serum LH was ≥10 IU/L. During the treatment, the Gn dose was adjusted according to the dynamically monitored follicle size, number and serum estradiol (E_2_) level until the follicles were mature and met the triggering criteria.

## 3. Progestin-primed ovarian stimulation protocol

Ovarian stimulation was initiated on menstrual cycle day 2 to 3. Based on the patient’s age, BMI, AMH, basal FSH, and AFC, Oral progesterone soft capsules (Utrogestan, 100 mg per capsule; Besins Healthcare, Belgium) were administered at a dose of 0.2 g/day concurrently with Gn at a daily dose of 150 to 300 IU. The Gn dose was subsequently adjusted according to serial monitoring of follicular size, number, and serum estradiol (E_2_) levels until follicular maturity was achieved and the criteria for trigger injection were met.

## 4. Trigger day and oocyte retrieval

The trigger injection was administered when at least 2 leading follicles reached ≥18 mm in diameter, 3 follicles ≥17 mm, or 2-thirds of follicles ≥16 mm. In the long follicular-phase protocol, a single trigger was used: human chorionic gonadotropin (hCG, 5000IU injection, Livzon Pharmaceutical Group Inc., Zhuhai, China) 7000 to 10,000 IU.

For the antagonist protocol and PPOS protocol, a dual trigger was employed: hCG 7000 IU combined with gonadotropin-releasing hormone agonist (GnRH-a; Triptorelin acetate 0.1 mg, Diphereline, Ipsen Pharma, France). Oocyte retrieval was performed under transvaginal ultrasound guidance 36 to 37 hours after trigger injection.

## 5. Embryo culture and transplantation

After oocyte retrieval in the fresh cycle, fertilization is performed by IVF or ICSI according to the patient’s clinical condition and prior treatment outcomes. On days 3 to 5 post-retrieval, 1 to 2 cleavage-stage embryos or blastocysts are selected for transfer. Considering the patient’s overall status in the current cycle and her preferences, either fresh ET or whole-embryo cryopreservation is performed. Patients treated with the PPOS protocol undergo whole-embryo cryopreservation.

## 6. Luteal support

Luteal-phase support was provided on the day of oocyte retrieval by oral administration of 10mg of a progestogen (dydroprogesterone 10 mg tablets for oral use; Duphaston®; Abbott Biologicals B.V.) every 12 hours and 2mg of an estrogen preparation (estradiol valerate 2 mg tablets for oral use; Progynova®; Merck Serono Europe Ltd., UK)every 12 hours, followed by a vaginal progesterone preparation (progesterone 90 mg gel for vaginal use; Crinone; Merck Serono Europe Ltd., UK) of 90mg of progesterone gel for on the day following oocyte retrieval. Clinical pregnancy was defined as pregnancy when gestational sac was detected by ultrasound 33 to 35 days after transplantation.

## 7. Outcome indicators

The study’s primary outcomes were the implantation rate (IR), clinical pregnancy rate (CPR), miscarriage rate (MR) per pregnancy, and LBR per ET. Oocyte maturation rate was calculated as the proportion of MII-stage oocytes to total retrieved oocytes. Fertilization was assessed 16 to 18 hours post-insemination by the presence of 2 pronuclei and 2 polar bodies. CPR was defined as the ultrasound detection of at least 1 gestational sac between the 5th and 6th weeks post-ET. IR was the ratio of gestational sacs to transferred embryos. MR was the spontaneous loss of all intrauterine pregnancies before 20 weeks of gestation. LBR was the number of live births after 20 weeks of gestation per ET.

## 8. Statistical methods

Statistical analyses were performed using SPSS version 26.0. Results, reported as counts (percentages), were compared using the χ^2^ or Fisher exact test. Continuous variables were tested for normality (Kolmogorov–Smirnov) and homogeneity of variance (Levene test). Normally distributed data (mean ± SD) were analyzed with Student *t* test or ANOVA; Univariate logistic regression was used for preliminary screening (*P* <.10). Multivariable analysis was conducted using backward stepwise logistic regression (removal at *P* >.10). Multicollinearity was assessed by VIF. Model fit was evaluated by the Hosmer-Lemeshow test. *P* <.05 was considered statistically significant.

## 9. Result

### 9.1. *Baseline characteristics of Groups A and B under the same protocol* (Table 1)

Age <35 years group: AFC in low-reserve group (A1) was significantly lower than that in normal reserve group (B1)(*P* = .0221), and there was no statistical difference in other indexes (age, BMI, infertility time, AMH).

**Table 1:** Baseline data for Group A and Group B.

Parameters	A1 (<35 ) (N = 70)	B1 (<35 ) (N = 1044)	*P*	A2 (≥35 ) (N = 25)	B2 (≥35 ) (N = 292)	*P*
Age (years)	30.44 ± 2.63	30.46 ± 2.55	.9538	36.52 ± 2.00	36.61 ± 1.61	.8203
BMI (kg/m^2^)	21.77 ± 0.35	21.76 ± 2.81	.9845	22.50 ± 0.71	22.61 ± 2.72	.8815
Infertility duration (year)	2.52 ± 0.14	2.45 ± 0.38	.8303	2.93 ± 0.12	3.19 ± 0.19	.6492
AMH (ng/mL)	1.03 ± 0.51	4.18 ± 0.47	.6767	0.91 ± 0.12	2.21 ± 0.53	<.0001
AFC	8.13 ± 4.88	9.11 ± 3.39	.0221	7.60 ± 2.83	8.60 ± 3.86	.2022
Infertile type (%) primary infertility	43 (61.43%)	636 (60.91%)	–	10 (40.00%)	122 (41.78%)	–
Secondary infertility	27 (38.57%)	408 (39.09%)	–	15 (60.00%)	170 (58.22%)	–

Continuous variables are presented as mean ± standard deviation; categorical variables as number (percentage). *P*-values indicate between-group comparisons.

AFC = antral follicle count, AMH = anti-Müllerian hormone, BMI = body mass index.

Age ≥35 years group: AMH in low-reserve group (A2) was significantly lower than that in normal reserve group (B2), and other indicators (age, BMI, infertility time, AFC) had no statistical difference.

### 9.2. *Ovulation induction and laboratory indicators of Groups A and B under the same protocol* (Table 2)

<35 years old group: hE2 level, Gn days, number of punctured follicles, number of retrieved oocytes, number of 2PN and number of good embryos were significantly lower in low-reserve group (A1) than in normal reserve group (B1)(all *P* <.05).

**Table 2:** Comparison of the results of Group A and Group B.

	A1 (<35)	B1 (<35)	*P*	A2 (≥35)	B2 (≥35)	*P*
	(N = 70)	(N = 1044)		(N = 25)	(N = 292)	
Basal FSH	6.11 ± 3.12	5.57 ± 2.13	.1377	6.71 ± 2.79	5.38 ± 3.36	.1137
Basal E2	22.60 ± 10.32	22.24 ± 16.51	.9094	17.52 ± 17.71	19.08 ± 14.52	.5735
Basal LH	2.99 ± 0.06	2.76 ± 1.99	.5143	2.32 ± 3.35	2.57 ± 1.99	.5617
Basal P	0.98 ± 0.07	0.68 ± 0.50	.1949	0.68 ± 0.35	0.63 ± 0.38	.6073
E2 on hCG day	1731 ± 660.51	2197 ± 1120.33	.0013	1585 ± 1128.09	1967 ± 1025.85	.1154
LH on hCG day	1.62 ± 0.33	1.44 ± 1.70	.4679	1.63 ± 1.04	1.29 ± 0.69	.1137
P on hCG day	0.88 ± 0.23	0.87 ± 0.52	.871	0.75 ± 0.37	0.83 ± 0.58	.3033
Gn duration	10.61 ± 2.39	11.27 ± 2.18	.0272	10.60 ± 1.87	11.25 ± 2.10	.1113
Total Gn dosage	1914 ± 644.32	1778 ± 517.27	.0863	1889 ± 435.96	1891 ± 521.96	.9818
No. of follicles punctured	9.16 ± 4.29	11.34 ± 4.92	.0001	7.72 ± 4.22	10.26 ± 4.21	.0073
Oocyte retrieved	7.69 ± 4.08	9.37 ± 4.40	.0014	6.16 ± 3.65	8.377 ± 3.67	.0069
Retrieved oocyte (%)	83.93%	82.61%	.4215	79.79%	81.61%	.5045
IVF	46	639	–	15	162	–
ICSI	24	405	–	10	130	–
MII rates (%)	77.40%	80.94%	.2422	93.75%	81.73%	.0109
2PN fertilized oocytes	4.73 ± 2.85	5.83 ± 3.29	.0026	4.16 ± 2.97	5.38 ± 2.93	.0588
High-quality embryos	2.26 ± 2.02	3.15 ± 2.50	.0007	2.32 ± 2.15	2.79 ± 2.14	.3028
Cleavage-stage embryo transfer	48	784	–	18	239	–
Blastocyst transfer	16	178	–	3	32	–
Average no. of Embryos transferred	1.73 ± 0.45	1.74 ± 0.44	.86	1.81 ± 0.40	1.86 ± 0.37	.6125

Continuous variables are presented as mean ± standard deviation; categorical variables as number or percentage. *P*-values indicate between-group comparisons.

2PN = 2 pronuclei, E2 = estradiol, FSH = follicle-stimulating hormone, Gn = gonadotropin, ICSI = intracytoplasmic sperm injection, IVF = in vitro fertilization, LH = luteinizing hormone, MII = metaphase II oocyte, P = progesterone.

≥35 years old group: The number of follicles punctured and oocytes retrieved in low-reserve group (A2) was significantly lower than that in normal reserve group (B2)(*P* <.05). In addition, the MII egg rate in A2 group was significantly higher than that in B2 group (*P* = .0109).

No significant difference was observed between the 2 groups regarding egg recovery rate and the average number of transferred embryos. In women under 35, the low-reserve group exhibited significantly fewer 2PN and good embryos, a trend not seen in women aged 35 and older. These findings indicate that the impact of decreased ovarian reserve on ovulation induction outcomes is age-specific: younger women with low-reserve show poorer performance on embryo laboratory indicators (2PN and good embryo counts), whereas older women with low-reserve have a higher MII egg rate, albeit with fewer follicles and retrieved eggs compared to those with normal reserve.

### 9.3. *Pregnancy outcomes of Groups A and B under the same protocol* (Table 3)

IRs, biochemical pregnancy rates, CPRs, abortion rates, ectopic pregnancy rates, and LBRs showed no statistically significant differences between LOR (group A) and NOR (group B) across age stratification (<35 years or ≥35 years)(all *P* >.05).

**Table 3:** Comparison of clinical outcomes between the 2 groups.

	<35 )		FISH *P*	≥35		FISH
	A1 (N = 70)	B1 (N = 1044)		A2 (N = 25)	B2 (N = 292)	*P*
Implantation	58.56%	51.25%	.1422	42.11%	44.33%	.8663
Rate (%)						
HCG positive rate (%)	75.00%	69.20%	.4015	76.19%	71.59%	.8032
Clinical	70.31%	67.98%	.7824	57.14%	62.73%	.6443
Pregnancy (%)						
Miscarriage	4.44%	6.73%	.7603	8.33%	15.88%	.6954
Pregnancy (%)						
Ectopic pregnancy (%)	0%	0.61%	.5988	0%	0.59%	.7899
Live birth (%)	63.51%	61.59%	.8057	52.17%	50.79%	.8979

Data are presented as percentage (%); *P*-values indicate between-group comparisons.

HCG = human chorionic gonadotropin.

Although the abortion rate in the low-reserve group was numerically lower in the ≥35 years group than in the normal-reserve group (8.33% vs 15.88%), the difference did not reach statistical significance (*P* = .6954).

Ovarian reserve status (low vs normal) had no significant effect on IR, biochemical pregnancy rate, CPR, abortion rate, ectopic pregnancy rate, and LBR, and these results were consistent among different age groups, indicating that ovarian reserve status did not significantly change the key outcomes of assisted reproduction.

### 9.4. *Baseline characteristics of Group A, Group C, and Group D* (Table 4)

Age <35 years group: no statistically significant differences were observed among groups A1, C1, and D1 in terms of age, BMI, or duration of infertility (*P* >.05). However, significant differences were found in AMH and AFC levels among the 3 groups (*P* <.0001), with group A1 showing the highest values.

**Table 4:** Baseline data of Group A, Group C, and Group D.

Parameters	<35			*P*-value	≥35			*P*-value
	A1 (N = 70)	C1 (N = 92)	D1 (N = 312)		A2 (N = 25)	C2 (N = 108)	D2 (N = 366)	
Age (year)	30.44 ± 2.63	30.84 ± 2.48	30.81 ± 2.43	.5371	36.52 ± 2.00	39.06 ± 3.40	38.01 ± 8.27	.0056
BMI (kg/m^2^)	21.77 ± 0.35	21.88 ± 3.70	21.84 ± 2.99	.9675	22.50 ± 0.71	22.18 ± 2.75	22.79 ± 4.52	.2942
Infertility duration (year)	2.52 ± 0.14	1.76 ± 1.74	2.16 ± 2.23	.3015	2.93 ± 0.12	3.56 ± 4.02	2.71 ± 3.20	.1899
AMH (ng/mL)	1.03 ± 0.51	0.79 ± 0.31	0.84 ± 0.24	<.0001	0.91 ± 0.12	0.75 ± 0.33	0.79 ± 0.26	.0469
AFC	8.13 ± 4.88	6.02 ± 3.50	5.61 ± 2.72	<.0001	7.60 ± 2.83	4.89 ± 2.87	4.91 ± 2.31	.0013
Infertile type(%)	–	–	–	–	–	–	–	–
Primary infertility	43 (61.43%)	56 (60.87%)	179 (57.37%)		11 (44.00%)	30 (27.78%)	108 (29.51%)	
Secondary infertility	27 (38.57%)	36 (39.13%)	133 (42.63%)	–	14 (56.00%)	78 (72.22%)	258 (70.49%)	–

Continuous variables are presented as mean ± standard deviation; categorical variables are presented as number (percentage). *P*-values indicate between-group comparisons.

AFC = antral follicle count, AMH = anti-Műllerian hormone, BMI = body mas index.

Age ≥35 years group: a statistically significant difference was noted in age distribution among groups A2, C2, and D2 (*P* = .0056), while no significant differences were observed in BMI or duration of infertility (*P* >.05). Consistent with the trend in the younger subgroup, significant differences were also detected in AMH (*P* = .0469) and AFC (*P* = .0013) among the 3 groups.

### 9.5. *Ovulation induction and laboratory indicators of Group A, Group C, and Group D)* (Table 5)

Group A demonstrated superior basal ovarian reserve markers, with significantly lower basal FSH, E2, and LH levels compared to Groups C and D (all *P* <.0001). During stimulation, Group A exhibited the highest hE2 levels and significantly higher numbers of Gn days, punctured follicles, retrieved oocytes, and 2PN zygotes compared to Groups C and D (*P* <.0001 and *P* = .0003, respectively).

**Table 5:** Ovulation induction and laboratory indicators of Group A, Group C, and Group D.

	<35岁			*P*值	≥35岁			*P*值
	A1 (N = 70)	C1 (N = 92)	D1 (N = 312)		A2 (N = 25)	C2 (N = 108)	D2 (N = 366)	
Basal FSH	6.11 ± 3.12	8.18 ± 3.91	8.64 ± 2.67	<.0001	6.71 ± 2.79	8.49 ± 5.90	8.72 ± 3.40	<.0001
Basal E2	22.60 ± 10.32	44.34 ± 59.40	35.77 ± 69.94	<.0001	17.52 ± 17.71	61.17 ± 69.47	42.09 ± 85.25	<.0001
Basal LH	2.99 ± 0.06	3.84 ± 1.68	3.80 ± 1.55	<.0001	2.32 ± 3.35	4.02 ± 3.91	3.87 ± 1.75	<.0001
Basal P	0.98 ± 0.07	1.39 ± 2.53	0.81 ± 0.87	.1708	0.68 ± 0.35	1.30 ± 2.29	1.00 ± 2.25	.73
E2 on hCG day	1731 ± 660.51	1166.28 ± 814.05	1464.17 ± 830.88	.0008	1585 ± 1128.09	967.23 ± 699.44	1240.19 ± 745.35	<.0001
LH on hCG day	1.62 ± 0.33	2.86 ± 1.95	4.44 ± 4.29	<.0001	1.63 ± 1.04	4.40 ± 4.49	4.65 ± 2.65	<.0001
P on hCG day	0.88 ± 0.23	1.08 ± 1.00	6.80 ± 4.87	<.0001	0.75 ± 0.37	0.85 ± 0.71	6.23 ± 4.81	<.0001
Gn duration	10.61 ± 2.39	9.25 ± 2.53	8.65 ± 1.70	<.0001	10.60 ± 1.87	7.96 ± 3.06	8.60 ± 1.75	<.0001
Total Gn dosage	1914.34 ± 644.32	1932.61 ± 772.12	1824.24 ± 462.19	.5679	1889 ± 435.96	1621.18 ± 745.79	1835.48 ± 459.54	.0007
No. of follicles punctured	9.16 ± 4.29	6.61 ± 5.34	6.33 ± 3.63	<.0001	7.72 ± 4.22	4.47 ± 3.08	5.11 ± 2.84	.0001
Oocyte retrieved	7.69 ± 4.08	5.26 ± 4.06	5.02 ± 3.10	<.0001	6.16 ± 3.65	3.64 ± 2.80	4.02 ± 2.44	.0007
IVF	46	61	182	–	15	59	197	–
ICSI	24	31	130	–	10	49	169	–
2PN fertilized oocytes	4.73 ± 2.85	3.34 ± 2.54	3.29 ± 2.36	.0003	4.16 ± 2.97	2.34 ± 2.26	2.56 ± 1.96	.0005
High-quality embryos	2.26 ± 2.02	2.18 ± 2.07	1.89 ± 1.67	.3955	2.32 ± 2.15	1.36 ± 1.58	1.59 ± 1.48	.0305
Cleavage-stage embryo transfer	48	8	0	–	18	6	0	–
Blastocyst transfer	16	0	0	–	3	0	0	–
Average no. of embryos transferred	1.73 ± 0.45	1.87 ± 0.35	0	.5427	1.81 ± 0.40	1.50 ± 0.58	0	.3871

Continuous variables are presented as mean ± standard deviation; categorical variables as number or percentage. *P*-values indicate between-group comparisons.

E2 = estradiol, FSH = follicle-stimulating hormone, Gn = gonadotropin, hCG = human chorionic gonadotropin, ICSI = intracytoplasmic sperm injection, IVF = in vitro fertilization, LH = luteinizing hormone,.

Stratified analysis by age revealed no significant differences in the number of high-quality embryos among groups in patients aged <35 years. However, in the ≥35 age group, Group A achieved the highest number of high-quality embryos (*P* = .0305), while Group C had the lowest total Gn dosage (*P* = .0007). Consistent with established consensus, clinical outcomes (oocytes retrieved, 2PN, and high-quality embryos) were generally lower in the ≥35 age group compared to the younger cohort.

### 9.6. *Factors associated with Clinical Pregnancy* (Table 6)

Logistic regression analysis was performed to evaluate predictors of clinical pregnancy. Univariate analysis identified age (*P* = .003), AFC(*P* <.001), and infertility type (*P* = .026) as significant factors, whereas AMH (*P* = .135) and other parameters showed no significant correlation.

**Table 6:** Univariate and multivariate logistic regression analysis of factors associated with clinical pregnancy (Group A and Group B).

Variables	Univariate analysisOR (95% CI)	*P*-value	Multivariate analysis*aOR (95% CI)	*P*-value
Age (year)	0.955 (0.926–0.985)	.003	0.952 (0.922–0.983)	.003
AFC	1.075 (1.038–1.113)	<.001	1.069 (1.032–1.107)	<.001
Infertility type	0.782 (0.630–0.971)	.026	0.734 (0.587–0.919)	.007
AMH (ng/mL)	1.158 (0.955–1.404)	.135	–	–
BMI (kg/m^2^)	1.003 (0.967–1.040)	.878	–	–
Basal E2 (pg/mL)	0.998 (0.996–1.001)	.207	–	–
Basal FSH (IU/L)	1.019 (0.982–1.058)	.321	–	–
Basal LH (IU/L)	1.030 (0.986–1.076)	.182	–	–
7Basal P(ng/mL)	0.959 (0.845–1.088)	.513	–	–
Gn duration (days)	1.039 (0.989–1.092)	.125	–	–
E2 on hCG day	1.000 (1.000–1.000)	.062	–	–
P on hCG day	0.969 (0.807–1.164)	.740	–	–
LH on hCG day	0.978 (0.884–1.082)	.664	–	–

The multivariate model was adjusted for Age, AFC, and infertility type (AMH and E2 were excluded as per the optimized model).

AFC = antral follicle count, AMH = anti-Műllerian hormone, aOR = adjusted odds ratio, BMI = body mass index, CI = confidence interval, E2 = estradiol, FSH = follicle-stimulating hormone, Gn = gonadotropin, hCG = human chorionic gonadotropin, LH = luteinizing hormone, OR = odds ratio.

* Adjusted odds ratio (aOR) from multivariate logistic regression, adjusted for confounders in the model. aOR > 1 indicates increased odds of outcome; aOR < 1 indicates reduced odds. 95% CI not containing 1 and *P* < .05 denote statistically significant associations.

In the multivariate model, Age, AFC, and Infertility Type remained independent predictors. Increasing age was negatively associated with pregnancy odds (adjusted odds ratio [aOR] = 0.952, 95% CI: 0.922–0.983, *P* = .003). Conversely, higher AFC significantly improved outcomes (aOR = 1.069, 95% CI: 1.032–1.107, *P* <.001). Infertility type was also independently associated with clinical pregnancy (aOR = 0.734, 95% CI: 0.587–0.919, *P* = .007).

### 9.7. *Factors associated with Live Birth* (Table 7)

Binary logistic regression analysis was conducted to evaluate predictors of live birth. Univariate analysis revealed that age (*P* <.001), AFC(*P* = .026), AMH(*P* = .015), Gn duration (*P* = .008), and hormone levels on hCG day (E2 and P) were significantly associated with live birth outcomes.

**Table 7:** Univariate and multivariate logistic regression analysis of factors associated with live birth (Group A and Group B).

Variables	Univariate analysisOR (95% CI)	*P*-value	Multivariate analysis*aOR (95% CI)	*P*-value
Age (year)	0.945 (0.917–0.974)	<.001	0.940 (0.911–0.970)	<.001
AFC	1.038 (1.005–1.072)	.026	–	–
Infertility type	0.817 (0.663–1.008)	.060	0.740 (0.595–0.920)	.007
AMH (ng/mL)	1.267 (1.048–1.532)	.015	–	–
BMI (kg/m^2^)	1.011 (0.976–1.047)	.556	–	–
Basal FSH (IU/L)	0.993 (0.958–1.030)	.724	–	–
Basal LH (IU/L)	0.968 (0.928–1.010)	.136	–	–
Basal E2 (pg/mL)	0.997 (0.994–1.001)	.169	–	–
Basal P (ng/mL)	0.921 (0.804–1.056)	.239	–	–
Gn duration (days)	1.068 (1.017–1.120)	.008	–	–
E2 on hCG day	1.000 (1.000–1.000)	.018	1.000 (1.000–1.000)	.001
P on hCG day	0.757 (0.627–0.914)	.004	0.657 (0.533–0.810)	<.001
LH on hCG day	0.971 (0.877–1.075)	.570	–	–
Total Gn dose	1.000 (1.000–1.000)	.994	–	–
Infertility duration	1.006 (0.962–1.053)	.783	–	–

The multivariate model was adjusted for Age, AFC, and infertility type (AMH and E2 were excluded as per the optimized model).

AFC = antral follicle count, AMH = anti-Műllerian hormone, aOR = adjusted odds ratio, BMI = body mass index, CI = confidence interval, E2 = estradiol, FSH = follicle-stimulating hormone, Gn = gonadotropin, hCG = human chorionic gonadotropin, LH = luteinizing hormone, OR = odds ratio.

* Adjusted odds ratio (aOR) from multivariate logistic regression, adjusted for confounders in the model. aOR > 1 indicates increased odds of outcome; aOR < 1 indicates reduced odds. 95% CI not containing 1 and *P* < .05 denote statistically significant associations.

In the multivariate logistic regression model, Age, Infertility Type, E2 on hCG day, and P on hCG day were identified as independent predictors. Age showed a significant negative correlation with live birth (adjusted odds ratio [aOR] = 0.940, 95% CI: 0.911–0.970, *P* <.001). Similarly, elevated progesterone (P) levels on hCG day were independently associated with a reduced likelihood of live birth (aOR = 0.657, 95% CI: 0.533–0.810, *P* <.001). Infertility type (aOR = 0.740, *P* = .007) and E2 levels on hCG day (*P* = .001) also remained statistically significant in the final model. Notably, while ovarian reserve markers (AFC and AMH) were significant in the univariate analysis, they were not independent predictors after adjustment.

According to the logistic regression analysis in Tables 6 and 7, the increase in female age and the rise in progesterone (P) on the day of hCG are strong independent predictors of decreased LBRs. Although the number of follicles (AFC) and anti-Müllerian hormone (AMH) showed significance in univariate analysis, they were not independent predictors after multivariate adjustment. In contrast, infertility type and estradiol (E2) on the day of hCG remained significant in the final model.

## 10. Discussion

AMH serves as a crucial biomarker for assessing ovarian reserve function and is closely associated with the outcomes of ART. Decreased levels of AMH are associated with diminished ovarian responsiveness to Gn, resulting in a suboptimal response characterized by inadequate follicular development synchronization, leading to an elevated risk of cycle cancelation. The absence of universally accepted diagnostic criteria for DOR has prompted the establishment of a threshold value of 1.2 ng/mL for AMH in this study. This value aligns with the diagnostic criteria for poor ovarian response in the Bologna criteria (1.1 ng/mL)^[24]^ and the ESART study (1.3 ng/mL),^[25]^ meeting the criteria for a low-reserve population as outlined by the POSEIDON panel in 2016.^[26]^ The POSEIDON classification system categorizes ART patients with reduced ovarian reserve or atypical responses to gonadotropins into 4 groups,^[27]^ considering age, AMH levels, AFC, and the number of oocytes retrieved in the initial stimulation cycle. This stratification method has achieved global acceptance among reproductive specialists.^[28]^

Clinical data indicate that patients with DOR account for approximately 31% of those undergoing ART.^[29]^ Therefore, optimizing treatment strategies for this specific population is crucial to improving overall fertility treatment outcomes. However, there is currently ongoing debate regarding the selection of COS protocols for DOR patients. It is important to clarify that the choice of COS protocol directly impacts the ultimate success rate of in vitro fertilization and ET (IVF-ET). In IVF treatment, selecting an appropriate stimulation protocol to increase the number of oocytes retrieved and improve pregnancy and LBRs is key to achieving successful outcomes.

For the DOR population, commonly used clinical protocols include: gonadotropin-releasing hormone agonist protocols (luteal-phase long protocol and follicular-phase long protocol), antagonist protocol, PPOS protocol, and mild stimulation protocol, among others. The traditional view suggests that long follicular-phase protocol may have an excessive suppressive effect on DOR patients. However, recent studies have indicated that this protocol still holds application value under specific circumstances.

The long follicular-phase protocol operates through the dual actions of GnRH agonists. Initially, GnRH agonists bind to pituitary receptors, stimulating the release of stored gonadotropins and causing a transient surge in serum FSH and LH levels (known as the flare-up effect). Prolonged administration results in pituitary receptor desensitization and depletion due to the resistance of GnRH agonists to enzymatic degradation and their extended half-life. This leads to pituitary suppression, decreased endogenous gonadotropin production, and maintenance of low ovarian hormone levels (down-regulation mechanism). Research indicates that supplementing with exogenous gonadotropins following pituitary down-regulation enhances the synchronized growth and maturation of multiple follicles, augments oocyte yield, and enhances the success rate of IVF procedures. This aligns with the therapeutic objective of the POSEIDON stratification strategy, which aims to optimize follicular synchrony in individuals with a poor prognosis. Furthermore, GnRH agonists can enhance egg and embryo quality by reducing levels of inflammatory mediators such as vascular endothelial growth factor and interleukin-1 (IL-1).^[30]^

The long follicular-phase protocol effectively suppresses early LH surges, synchronizes follicular development, increases oocyte retrieval, reduces cycle cancelation rates, and maintains stable CPRs.^[31]^ In women with diminished ovarian reserve (DOR), the proportion of germinal vesicle and meiotic I (MI) oocytes is higher than in those with NOR, often due to asynchronous follicular recruitment.^[32,33]^ However, this protocol has limitations: low estrogen levels after pituitary down-regulation can cause perimenopausal symptoms such as hot flashes and irritability, there is a high incidence of ovarian hyperstimulation syndrome (OHSS), and it requires more gonadotropins, prolonging treatment duration and increasing costs.

The long follicular-phase protocol have been shown to enhance endometrial receptivity, optimize the pelvic microenvironment, and facilitate embryo implantation, ultimately leading to higher CPRs and decreased abortion rates.^[31]^ Research indicates that suppressing pituitary function through the administration of a full-dose slow-release gonadotropin-releasing hormone agonist (GnRH-a) in a prolonged follicular-phase protocol can improve endometrial receptivity during the implantation of embryos.^[23,31]^ Animal studies have further demonstrated that preimplantation embryos cultured in a GnRH-a enriched environment exhibit enhanced developmental capacity, with the expression of GnRH and its receptor detected in human preimplantation embryos.^[31,34]^ Markers such as homeobox gene (HOX) A10, pinocytin, and integrin have been identified as indicators of endometrial receptivity, showing a positive correlation with receptivity levels.^[35]^ GnRH-a treatment has been linked to increased pregnancy rates through elevated pinocyte numbers, enhanced integrin levels, and improved endometrial receptivity. Moreover, higher doses and longer durations of GnRH-a have been found to inhibit irregular endometrial growth, local inflammatory responses, autoantibody production, and reduce levels of tumor necrosis factor and interleukin-1^[35]^ in bodily fluids, all of which are conducive to successful embryo implantation.

Our study also found that for patients with LOR (AMH <1.2 ng/mL), the use of the long follicular-phase protocol in the first cycle, despite yielding fewer retrieved oocytes and high-quality embryos compared to those with NOR, still resulted in similar CPRs and LBRs. This may be attributed to the protocol’s ability to modulate the patient’s endocrine environment and improve endometrial receptivity. Within the low-reserve population, long follicular-phase protocol demonstrated superior performance in terms of the number of oocytes retrieved, fertilization capability, and formation of high-quality embryos compared to the antagonist protocol and the PPOS protocol.

Age serves as a core prognostic factor, independent of the influence of ovarian reserve markers. Our study provided strong support for this view through multivariate logistic regression analysis: after adjusting for confounding factors such as AFC, AMH, and hormone levels, age remained an independent negative predictor of clinical pregnancy and live birth outcomes. Previous studies have shown that younger women with DOR have higher pregnancy rates and relatively lower MRs, significantly outperforming their older counterparts.^[36–38]^ Our live birth model further confirmed this finding, as AMH – an indicator of ovarian reserve – was excluded from the multivariate analysis due to lack of statistical significance, while age consistently maintained high significance. This indicates that age-related decline in ovarian function is an important factor affecting reproductive outcomes in older pregnant women.

Based on the study findings, implementing a prolonged follicular-phase regimen for individuals with LOR (AMH <1.2 ng/mL) should adhere to the principle of personalized screening. For individuals under 35 years old with AFC of 5 or more, this regimen can be considered the primary option. It effectively suppresses the endogenous LH surge, enhances endometrial receptivity, and yields fresh ET pregnancy rates comparable to those with NOR. Conversely, for individuals aged 35 or older or with an AFC of <5, a preference for the “cumulative embryo strategy” is recommended. This approach involves accumulating embryos over multiple egg retrieval cycles for subsequent frozen ETs.

This retrospective study is subject to inherent limitations. Firstly, confounding factors such as lifestyle choices (e.g., smoking, sleep patterns) and underlying medical conditions (e.g., thyroid dysfunction) were not accounted for, potentially impacting the efficacy analysis. Secondly, the baseline data of different ovarian stimulation protocol groups in the low-reserve population are unbalanced. The baseline conditions of group A are better, and there is selection bias in protocol selection, which cannot completely rule out the interference of baseline differences on ovulation induction outcomes (such as the number of retrieved oocytes and the number of high-quality embryos). Thirdly, the extrapolation of single-center data is limited and requires verification through multi-center studies. In the future, preimplantation genetic testing (PGT-A) can be combined to clarify the impact of the long-action protocol in the follicular-phase on the embryo euploidy rate.^[39]^ In conclusion, the long follicular-phase protocol has selective application value in the low-reserve population with AMH <1.2 ng/mL, and its efficacy is regulated by age, AFC and POSEIDON subgroup characteristics. By precisely stratifying and screening patients, the advantages of the treatment plan can be maximized, providing individualized treatment options for people with a low prognosis.

## Acknowledgments

We appreciate the patient’s cooperation.

## Author contributions

**Data curation:** Fang Hong, Huaying Yu.

**Formal analysis:** Fang Hong, Feng Zhou.

**Writing – original draft:** Fang Hong.

**Writing – review & editing:** Fang Hong, Xiaomei Tong.
